# Development and Evaluation of an Ethical Guideline for Decisions to Limit Life-Prolonging Treatment in Advanced Cancer: Protocol for a Monocentric Mixed-Method Interventional Study

**DOI:** 10.2196/resprot.9698

**Published:** 2018-06-15

**Authors:** Katsiaryna Laryionava, Katja Mehlis, Elena Bierwirth, Friederike Mumm, Wolfgang Hiddemann, Pia Heußner, Eva C Winkler

**Affiliations:** ^1^ National Center for Tumor Diseases Department of Medical Oncology Heidelberg University Hospital Heidelberg Germany; ^2^ Interdisciplinary Center of Psycho-Oncology University Hospital Grosshadern Ludwig-Maximilians University Munich Germany; ^3^ Department of Medicine III University Hospital Grosshadern Ludwig-Maximilians University Munich Germany

**Keywords:** ethical guidelines, treatment limitation, advanced cancer

## Abstract

**Background:**

Many patients with advanced cancer receive chemotherapy close to death and are referred too late to palliative or hospice care, and therefore die under therapy or in intensive care units. Oncologists still have difficulties in involving patients appropriately in decisions about limiting tumor-specific or life-prolonging treatment.

**Objective:**

The aim of this Ethics Policy for Advanced Care Planning and Limiting Treatment Study is to develop an ethical guideline for end-of-life decisions and to evaluate the impact of this guideline on clinical practice regarding the following target goals: reduction of decisional conflicts, improvement of documentation transparency and traceability, reduction of distress of the caregiver team, and better knowledge and consideration of patients’ preferences.

**Methods:**

This is a protocol for a pre-post interventional study that analyzes the clinical practice on treatment limitation before and after the guideline implementation. An embedded researcher design with a mixed-method approach encompassing both qualitative and quantitative methods is used. The study consists of three stages: (1) the preinterventional phase, (2) the intervention (development and implementation of the guideline), and 3) the postinterventional phase (evaluation of the guideline’s impact on clinical practice). We evaluate the process of decision-making related to limiting treatment from different perspectives of oncologists, nurses, and patients; comparing them to each other will allow us to develop the guideline based on the interests of all parties.

**Results:**

The first preintervention data of the project have already been published, which detailed a qualitative study with oncologists and oncology nurses (n=29), where different approaches to initiation of end-of-life discussions were ethically weighted. A framework for oncologists was elaborated, and the study favored an anticipatory approach of preparing patients for forgoing therapy throughout the course of disease. Another preimplementational study of current decision-making practice (n=567 patients documented) demonstrated that decisions to limit treatment preceded the death of many cancer patients (62/76, 82% of deceased patients). However, such decisions were usually made in the last week of life, which was relatively late.

**Conclusions:**

The intervention will be evaluated with respect to the following endpoints: better knowledge and consideration of patients’ treatment wishes; reduction of decisional conflicts; improvement of documentation transparency and traceability; and reduction of the psychological and moral distress of a caregiver team.

**Registered Report Identifier:**

RR1-10.2196/9698

## Introduction

Decision-making to limit a tumor-specific and life-prolonging treatment in patients with advanced cancer is one of the most difficult tasks in end-of-life care and is often accompanied by psychological and ethical challenges. Many patients with advanced cancer receive aggressive chemotherapy close to death and are referred to palliative or hospice care too late, and die under therapy or in the intensive care unit [1-3]. Thus, the number of patients receiving chemotherapy between two weeks and death has been constantly rising [4]. The literature suggests that discussions about patients’ end-of-life preferences and treatment limitation should occur much earlier. Such discussions have been associated with better patient outcomes in terms of increasing patients’ quality of life and reducing patients’ anxiety, distress, and depression [3,5,6].

However, in a previous study we demonstrated that only 37 of 76 patients (47%) with incurable cancer had discussions about limiting treatment or got involved in decisions about limiting tumor-specific or life-prolonging treatment (eg, do-not-resuscitate orders) [7]. Oncologists still have difficulties in discussing treatment limitation with their patients and feel unsure about when and how to initiate such discussions [8,9]. Against this background, we initiated a monocentric interventional study aimed at developing an ethical guideline for end-of-life decision-making, with a goal to increase oncologists’ awareness of patients’ treatment preferences, to increase patients’ involvement in decision-making, and to reduce psychological and moral distress within a caregiver team. According to many evaluational studies, guidelines can change clinical practice and may lead to the improvement of physician performance and patient outcomes [10,11].

While there are many studies on impact and uptake of guidelines in medical practice, ethical guidelines remain underrepresented in research and the impact of such guidelines in medical practice is not well studied. In the following protocol, we explain our study design for evaluating the impact of ethical guidelines on the practice of clinical decision-making regarding treatment limitation. The overall aims of this three-stage interventional Ethics Policy for Advanced Care Planning and Limiting Treatment (EPAL) Study are: (1) to develop an ethical guideline for end-of-life decision making, and (2) to evaluate its impact on decision-making practice. The guideline will be evaluated with regard to the following outcomes: better knowledge and consideration of patients’ treatment wishes, reduction of decisional conflicts, improvement of documentation transparency and traceability, and reduction of the psychological and moral distress of a caregiver team. We also anticipate that the guideline will enable oncologists to make timelier and ethically informed treatment limitation decisions.

## Methods

### Design

This is a pre-post interventional design that analyzes the clinical practice of decision-making before and after the guideline implementation. The study consists of three stages: (1) the preinterventional phase (Time 1 [T1]; status quo), followed by the (2) intervention phase (development and implementation of the guideline; T2), and (3) the postinterventional phase (T3).

We use an embedded researcher design with a mixed-method approach that encompasses both qualitative and quantitative methods. We assume that using one research method is not sufficient to answer all of our research questions and that different methods are required. Embedded researcher design mixes different data sets at different design phases so that one data type provides a supportive, supplemental role or is embedded within other data types [12].

In phase 1 (T1), the current clinical practice of decision-making and documentation of decisions to limit treatment will be analyzed from three different perspectives: patients, their treating oncologists, and nurses. Phase 2 (T2) entails the development and implementation of a guideline on advanced care planning and limiting treatment. In phase 3 (T3), the impact of the guideline on the practice of decision making and documentation will be analyzed. The project outline is presented in Table 1. This study protocol has been approved by the Ethics Committee of the Medical Faculty of the University of Munich.

### Study Setting

The study is monocentric and is being carried out at the Department of Hematology & Medical Oncology at the University Hospital of Munich, Germany. A monocentric approach is being used, because the development of a guideline has proven to be more successful when it is developed within and for a particular location, taking into account specifics of the organizational context and of stakeholders’ positions and professional self-conception of that environment [10].

#### Phase 1 (T1): Preimplementational Study of Current Decision-Making Practice of Limiting Tumor-Specific or Life-Prolonging Treatment

The first phase consists of three studies in which different methods are applied, with the aim to analyze the current state of decisions to limit a tumor-specific and life-prolonging treatment and to understand patients’, oncologists’, and nurses’ needs regarding decisions to limit treatment. The insights from phase 1 will be used for development of the ethical guideline and for comparison with the results of phase 3 in order to detect any impact of the guideline after implementation in the pre-post comparison.

**Table 1:** Project outline.

Phases	Methods	Data collection	Sample/participants
Phase 1 (T1): Preinterventional phase – baseline survey	1. Embedded research	1. Documentation analysis	1. 500 patients’ records
	2. Qualitative study	2. Semistructured interviews	2. 25 oncologists and nurses
	3. Quantitative survey	3. Questionnaire	3. 60 patients’ cases (oncologists + nurses)
Phase 2 (T2): Intervention development and implementation of the ethical guideline	1. Group discussions with experts	N/A^a^	1. Oncologists, nurses, psycho-oncologists, external experts for palliative medicine, medical ethicists, medical law, ethics committee
	2. Several consensus conferences	N/A	N/A
Phase 3 (T3): Postinterventional phase – comparison with the results of phase 1 and evaluation	1. Embedded research	1. Documentation analysis	1. 500 patients’ records
	2. Quantitative survey	2. Questionnaire	2. 60 patient’s cases (oncologists and nurses)

^a^N/A: not applicable.

Phase 1 includes: an observational baseline-survey on documentation, frequency, and timing of decisions to limit intensive medical care and tumor-specific therapy; a qualitative interview study with oncologists and oncology nurses; and a quantitative survey with patients, nurses, and oncologists.

##### Observational Baseline-Survey on Frequency and Timing of Decisions to Limit Intensive Medical Care and Tumor-Specific Therapy

As a first step of the preimplementational phase, we aim to examine how often decisions to limit treatment precede patients’ deaths and how early they are determined. This baseline survey will enable us to investigate whether findings from the quantitative survey with oncologists, nurses, and patients are supported by observation and vice versa. The main research questions of the survey are presented in Textbox 1.

For the observation, we will use a standardized documentation form. This form will be developed in a multi-step team process that will encompass literature review and discussions with experts including oncologists, palliative care physicians, medical ethicists, and social science experts. The documentation form will include the following information: patients’ demographic data and diagnosis, the therapy/ intervention that have decided to be limited, how decisions to limit treatment are documented, changes in the scope of decisions to limit treatment, place of patient’s death, therapy and medication 72 hours before patient’s death, and whether decisions to limit treatment are followed or overruled in a patient’s last days.

###### Methods and Data Collection

We will use a method of an *embedded researcher* for this part of the project. The researcher is a member of a hospital team and participates in patients’ sign-out reports from late to night shifts. She documents all treatment decisions made by oncologists. However, her role remains a passive one in order to avoid any influence on the current situation. If a patient dies, we will document all relevant decisions that preceded death. Furthermore, patients’ transfer plans, charts, electronic hospital acts, and discharge letters will be analyzed.

###### Participants

All inpatients with advanced hematological/oncological neoplasia will be included if they are under treatment in at the Department of Hematology/Medical Oncology at the University Hospital in Munich.

###### Data Analysis

Data will be analyzed using Microsoft Excel.

##### Qualitative Interview Study With Oncologists and Oncology Nurses

In order to explore in-depth how oncologists and oncology nurses perceive treatment limitation decisions, a qualitative approach based on a grounded theory methodology will be used.

###### Methods and Data Collection

Qualitative individual face-to-face interviews will be conducted using a semistructured and pilot-tested interview guide. The interview topics are presented in Textbox 2.

**Research questions.**

- How often do decisions to limit treatment precede a patient’s death?
- How often do patients die under chemotherapy or in the intensive care unit?
- How intensely are patients treated shortly before death?
- How well are decisions to limit treatment documented?
- How long before patients' death are decisions to limit treatment made?

**Main interview topics.**

- Treatment limitation situations
- Decision-making process
- Patients’ involvement in decision-making
- Role of patients’ family in decision-making
- Nurses’ role in decisions to limit treatment
- Challenges and conflicts by decisions to limit treatment
- Oncologists’ role in decisions to limit treatment

###### Procedure and Data Analysis

Purposive and theoretical sampling strategies will be applied. Participants are purposely sampled to represent different hospital units, working experience, age, and sex to reflect a wide range of opinions. The sampling will be continued until the theoretical saturation will be reached: when no new categories emerge and the relationships among categories are well-developed [13].

The collected data will be analyzed using the three-stage approach (open, axial, and selective coding strategies) of grounded theory methodology. MAXQDA software (VER BI GmbH, Berlin, Germany) will be used to assist with the coding and management of transcripts.

#### Quantitative Survey With Patients, Nurses, and Oncologists

In order to access and compare the perspective and reports of parties involved in decisions to limit treatment, we will survey patients, their respective oncologists, and oncology nurses. The survey will be complemented by a documentation form completed by a project researcher on each patient’s case.

##### Patients’ Survey

###### Development of the Questionnaires

The study questionnaire will be developed based on a review of current literature and oncologists’ and nurses’ views and experiences derived from the interview study. A preliminary version of the questions will be checked by three experts (members of the research team) including an experienced psycho-oncologist, an oncologist with expertise in medical ethics, and a social scientist. We plan to use both established instruments as well as self-developed questions.

###### Established Instruments

The patients’ questionnaire set comprises the questionnaire on: distress in Cancer Patients [14]; Whooley Depression Scale [15]; the perceived patients’ quality of life, which will be measured by a quality of life scale from European Organization for Research and Treatment of Cancer Quality of Life Questionnaire-C30 [16]; and the trade-off between patients’ preferences for quality and length of life will be assessed with the Quality and Quantity Questionnaire [17]. We will translate and validate this questionnaire on a sample of advanced cancer patients within the scope of this project: the perceived role of patients’ families in decision-making will be measured with the scale *Family Involvement in Treatment Decisions* from the validated German version of the Cancer Communication Assessment Tool for Patients and Families [18], and patients’ roles in medical treatment decisions will be assessed with a German version of Control Preference Scale [19]. Satisfaction with a physician-patient interaction will be assessed using the validated questionnaire on the Quality of Physician-Patient Interaction [20]. We will use self-developed closed-ended questions with Likert scales to assess: patients’ awareness of the treatment goal (curative or palliative); patients’ recollection of discussing the treatment aim; patients’ information needs regarding disease, prognoses, advance directives, and actual information received by patients; patients’ communication preferences regarding treatment limitation; and satisfaction with decision-making.

###### Patient Recruitment

The inclusion criteria for patients are presented in Textbox 3.

We will exclude patients with cognitive impairment and/or with a very poor general state of health. Patients will be recruited through a project researcher who will identify patients with decisions to limit treatment. In addition, oncologists will refer eligible patients when treatment limitation has been/is being discussed.

Patients will be recruited from five hospital units (n=5 normal wards) and the recruitment will take place until 60 patients with decisions to limit treatment are included in the study. All patients matching the inclusion criteria will be contacted by a project oncologist who will inform them about study aims and content.

**Inclusion criteria.**

- Hospitalized cancer patients (male and female) over 18 years of age
- Diagnosed with advanced hematological/oncological neoplasia
- Decisions to limit treatment being either discussed or determined
- Adequate level of consciousness to complete the questionnaire
- Patients’ agreement through an informed consent document

**Table 2:** Topics addressed in the questionnaire.

Assessed topics	Patients	Oncologists	Nurses
Cancer-specific distress	✓	—	—
Depression	✓	—	—
Information needs regarding disease (eg, prognosis, side effects, life expectation)	✓	—	—
Actual received information regarding disease	✓	—	—
Factors that influenced oncologists’ decision of treatment limitation (eg, patients’ age, quality of life)	—	✓	—
Estimation of patients’ life expectation	—	✓	✓
Perceived (estimated) quality of life	✓	✓	✓
Information need for advanced directive/role of the advanced directive	✓	✓	✓
Discussed treatment limitation	✓	✓	✓
Planned or current treatment	✓	✓	✓
Level of difficulty of treatment limitation decisions	—	✓	—
Challenges that influenced treatment limitation	—	✓	✓
Consensus relating to treatment limitation decisions	—	✓	✓
Involvement of nurses in decision-making	—	✓	✓
Perceived aim of the treatment/estimation of patients’ preferences regarding treatment limitation	✓	✓	✓
Discussion of the treatment aim/involvement of patients in decision-making	✓	✓	✓
Preference for quality or length of life	✓	✓	✓
Role in medical decisions	✓	✓	✓
Estimation of awareness of patients’ prognosis	—	✓	✓
Satisfaction with oncologists’ communication/communication with patients	✓	✓	✓
Perceived role of the family, wish for family involvement/involvement of family members	✓	✓	✓
Satisfaction with treatment decisions/consensus with oncologist	✓	✓	✓
Perception of optimal time for treatment limitation	—	✓	✓
Moral distress and its reasons	—	✓	✓
Support needs	—	✓	✓

##### Oncologists’ and Nurses’ Survey

###### Development of the Questionnaires

The oncologists’ and nurses’ questionnaire set will be developed as analogues to the patients’ questionnaire to compare the answers to the same topics. In addition, we will assess moral distress in the care team with the Moral Distress Thermometer [21] and with two self-formulated open-ended questions on reasons for distress.

Furthermore, we will formulate questions to assess aspects such as: perceived difficulties by oncologists and nurses with decisions to limit treatment, oncologists’ and nurses’ satisfaction with decisions to limit treatment, and involvement of nurses in decisions to limit treatment. All topics that will be compared among oncologists, nurses, and patients are presented in Table 2.

####### Recruitment of Treating Physicians and Nurses at the Hematology/Oncology Inpatient Unit

For every patient’s case the respective oncologist in charge and oncology nurse will be surveyed. The respective treating oncologist of the selected patients will be contacted and asked to participate in the study.

####### Data Analysis

Patients’ characteristics will be evaluated using descriptive statistics. Patient cohorts will be formed based on patients’ preference for length or quality of life, specificity of decease type (ie, oncological/hematological disease), type of therapy, and psycho-social variables. To compare the oncologists’, nurses’, and patients’ views before and after guideline implementation, the Student t-test for paired data will be computed. Finally, linear regression analysis will be performed to identify possible predictors of oncologists’ decisions to limit treatment and patients’ involvement or noninvolvement in decision-making. Statistical significance will be assessed at the level of α=0.05 (two-sided). All statistical analyses will be conducted using SPSS v.20.

###### Additional Documentation on Every Patient’s Case

We will develop a special documentation form which will be completed by a project researcher on every patient’s case, in addition to the quantitative survey. The aim of this form is to gather additional objective information regarding treatment limitation decisions. The researcher will complete the documentation form as soon as the patient has completed the questionnaire (date 1) and will proceed with documentation seven days later (date 2). If the decision has not been made yet, the documentation form will be filled approximately three days later, after date 2 once again (date 3).

**Source of Information**

The project researcher will screen patients’ medical records to get necessary patient information. The documentation form will include information regarding hospital unit, patients’ diagnosis and performance status (*Eastern Cooperative Oncology Group* Performance Status), patients’ distress level at the time of hospital admission, pain information (numerical rating scale), type of therapy (eg, tumor-specific therapy [curative/palliative], anti-infective, radiation, parenteral nutrition, intravenous fluid substitution, transfusion, long-term medication for secondary diagnosis), and what additional supportive medication (eg, analgesics, sedatives) patients receive at the moment of documentation, as well as the availability of advance directives.

Additionally, the documentation form aims to assess other information, such as: patients’ wishes regarding decisions to limit treatment, and if and where it is documented; if decisions to limit treatment have been made, and if and where they are documented; and, if a palliative consultation, if psycho-oncological consultation / support are offered and if social services are involved. All changes regarding treatment limitation in the next week will be noted as well.

#### Phase 2 (T2): Development and Implementation of the Guideline

The guideline will be developed in four interprofessional working groups with equal participation of clinic management, senior and assistant physicians, psycho-oncologists, nursing management, nurses, and quality managers. In group settings the participants will discuss different topics related to treatment limitation, communication, documentation of decisions, and legal aspects. Each group will formulate statements and recommendations which will be finally approved by the mandate holders that are eligible to vote. Finally, the guideline will be presented to the external experts of palliative medicine, medical ethics, and medical law for editing and improvement. The methodology of guideline development will be described in detail in a separate paper.

##### The Intervention

After presentation of the guideline at the internal hospital conference, mandatory training courses will be offered for oncologists and nurses to become familiar with the application of the guideline in their daily practice.

#### Phase 3 (T3): Postinterventional Study of Decision-Making Practice

After implementation of the guideline, all measurements from phase 1 (T1), aside from the in-depth qualitative interviews with oncologists and nurses, will be repeated to assess possible changes in clinical practice after guideline implementation.

##### Measure of Compliance to the Guideline

Oncologists’ self-reported compliance to the guideline will be measured using several additional self-formulated questions on oncologists’ guideline adherence that will be placed at the end of the questionnaire in phase 3. These questions will assess if the guideline will be used by oncologists in every patient’s case and whether it will be helpful for end-of-life decision making. Furthermore, a short additional questionnaire is planned to assess oncologists’ opinions on the applicability and practicability of the implemented guideline.

## Results

Some parts of the project have been completed and published [17,22]. A qualitative study with 29 oncologists and oncology nurses revealed that participants had different approaches to initiation of end-of-life discussions. These approaches were ethically weighted and a framework for oncologists was elaborated. This framework favored an anticipatory approach of preparing patients for forgoing therapy throughout the course of disease [22]. The preimplementation study of current decision-making practice (n=567 patients documented) demonstrated that decisions to limit treatment preceded the death of many patients with a cancer disease (62/76, 82% of deceased patients), but usually were made in the last week of life [17].

## Discussion

### Overview

This paper describes the study design of an implementation and evaluation strategy of an ethical guideline for decision-making related to limiting treatment in advanced cancer patients that is grounded in empirical data. The pursued goal is to provide an informed, transparent, and ethically founded approach to controversial questions associated with treatment limitation, taking into account a specific environment of a university hospital.

As far as we are aware, this is the first study of its kind that uses a mixed-method approach, and involves patients, oncologists, and nurses to evaluate the impact of an ethical guideline on clinical practice. According to the literature review on institutional ethics policies for end-of-life decisions conducted by Lemiengre et al [23], most studies have focused on the implementation of do not-resuscitate policies; however, the use of before-and-after designs is scarce. Some studies deal with policies on pain, symptom control, and euthanasia [23]. However, there is a lack of pre-post interventional studies on ethical guideline development.

### Study Strengths

#### Analysis of Different Perspectives

One major strength of this study is the evaluation of decision-making related to limiting treatment from different perspectives of oncologists, nurses, and patients, and comparing them to each other before and after implementation of the guideline. Additionally, we will analyze oncologists’, nurses’, and patients’ needs and perceived difficulties in decision-making that will allow us to develop a guideline based on the interests of all parties. Furthermore, we will assess the perspective and needs of patients with advanced cancer shortly before dying. This patient group is not easy to include in studies as they are very weak and need a sensitive approach when they are assessed on end-of-life topics.

#### Mixed-Method Approach

An important strength of this study is the application of different methods and the collection of multiple sources of data (interviews, observations, and surveys) and perspectives of different parties (patients, oncologists, and nurses) to study the same topic. The triangulation of data contributes to the credibility of the results and will make the findings more grounded, thereby offsetting the weaknesses of both quantitative and qualitative research [12].

#### Multidisciplinary Team

For all phases of the project we will engage with a multidisciplinary team that includes experienced experts from psycho-oncology, sociology, clinical oncology, nursery, palliative medicine, and medical ethics. Decision-making at the end of life is a complex process that faces challenges of psychological, medical, and ethical natures. Different professional backgrounds and skills could provide a better framework for analysis and understanding of this process.

### Study Limitations

One of the considerable limitations is a well-documented challenge that is associated with the pre-post interventional design studies. Pre-post studies assume that any difference in measurement in “prestudy” compared with “poststudy” is due to the intervention; however, they do not account for other elements that are also changing at the same time that the intervention is taking place. It is difficult to determine if a certain intervention has indeed produced observed improvement.

A further limitation is that we have to use different patients’ samples in pretests and posttests. The development and implementation of the guideline will take some time and many patients will not be available to participate in the posttest measurements. Furthermore, we must consider that by partaking in in-depth qualitative interviews and a very extensive questionnaire, the awareness of ethical issues among oncologists and nurses could be considerably raised. Consequently, it is difficult to distinguish the effects of the study itself on the clinical practice of decision-making from the effects caused by the guideline implementation. However, we should note that there is a high rate of fluctuation in a university hospital and this argument might not apply.

Additionally, the observed improvement could be due to the so-called Hawthorne effect (or observer effect) when participants change or adapt their behavior due to the awareness of observation and assessment [24]. This effect is not easy to quantify. However, literature suggests that triangulation of data using a variety of methods from different sources can contribute to reducing or even overcoming the mentioned effect [25,26]. A further limitation is related to certain particularities of a large university hospital setting. A characteristic of a university hospital environment is that junior staff at the wards rotate every 3-6 months. Consequently, some junior oncologists that participate in the preintervention phase may not have been present at the guideline implementation phase as well as at the postevaluation phase.

### Conclusion

The results of this study aim at improving the development and implementation of ethical guidelines into clinical practice. We expect that our intervention will contribute to improvements in the decision-making processes on treatment limitation in patients with advanced cancer, increase the awareness for patient treatment preferences and their involvement in decision-making, and reduce psychological and moral distress within the caregiver team. Another expected outcome of the intervention is improvements in documentation transparency and traceability.

## Appendix

Multimedia Appendix 1 Peer-reviewer report (German).

## Abbreviations

EPAL: Ethics Policy for Advanced Care Planning and Limiting Treatment
