# Supplementary material for: Development and Evaluation of an Ethical Guideline for Decisions to Limit Life-Prolonging Treatment in Advanced Cancer: Protocol for a Monocentric Mixed-Method Interventional Study
Source: JMIR Res Protoc. 2018 Jun 15;7(6):e157. doi: 10.2196/resprot.9698 (PMC6026302; doi:10.2196/resprot.9698)
Supplement: Multimedia Appendix 1 [file resprot_v7i6e157_app1.pdf]

Quintessenzen aus zwei Gutachten zum Antrag 109658

Gutachter 1

"... Anregungen zur Projektplanung:

- Durchführung einer systematischen Recherche nach bereits existierenden Leitlinien zum Thema in einschlägigen Datenbanken (nicht nur PubMed) als Ausgangspunkt der Entwicklung einer lokalen Leitlinie.
- Präzisierung der Einschlusskriterien für Patienten.
- Erfassung verstorbener Patienten: Dokumentation der Todesursache.
- Klärung der in Frage stehenden Entscheidungssituation für den Patienten: Trade-off zwischen Verlängerung der Lebenszeit und Einschränkung der Lebensqualität oder Nicht-Erwartung einer Verlängerung der Lebenserwartung und/oder Nichterwartung einer Verbesserung der Lebensqualität und/oder Einschränkung der Lebensqualität durch Fortführung einer tumorgerichteten Therapie?
- Berücksichtigung des Rechts auf 'Nicht-Wissen-Wollen' in spezifischen Situationen.
- Überdenkung des Studiendesigns (Cluster-Randomisierung? Konsekutiv rekrutierte prospektive Kohortenstudie mit Kontrollgruppe?) ..."

Gutachter 2

"... Der Aufbau des Arbeitsprogramms ist schlüssig. Allerdings fehlt eine Stellungnahme der Antragsteller, ob diejenigen Patienten dokumentiert und ausgewertet werden, die eine Studienteilnahme ablehnen. Weiterhin bleibt unklar, in wieweit die Angehörigen direkt oder indirekt mit in die Leitlinie einbezogen werden. ..."
